# A Single Lesson on Dietary Education Improves Dietary Knowledge in Adults with Type 2 Diabetes: A Real-Life Monocentric Italian Study

**DOI:** 10.3390/nu17071139

**Published:** 2025-03-26

**Authors:** Olga Eugenia Disoteo, Federica Russo, Luigi Renzullo, Giulia Negri, Giuseppina Piazzolla, Giovanni De Pergola, Vincenzo Triggiani, Giuseppe Lisco

**Affiliations:** 1 Diabetes Unit, Niguarda Cà Granda Hospital, 20162 Milan, Italy; olgaeugenia.disoteo.amediabete@gmail.com (O.E.D.); dottoressarussofederica@gmail.com (F.R.); luigi.renzullo92@gmail.com (L.R.); nutrizionistanegri@gmail.com (G.N.); 2 Division of Endocrinology, Diabetology and Clinical Nutrition, Sant’Anna Hospital—ASST Lariana, 22042 Como, Italy; 3 Interdisciplinary Department of Medicine, School of Medicine, University of Bari “Aldo Moro”, Piazza Giulio Cesare 11, 70124 Bari, Italy; giuseppina.piazzolla@uniba.it (G.P.); giuseppe.lisco@uniba.it (G.L.); 4 Center of Nutrition for the Research and the Care of Obesity and Metabolic Diseases, National Institute of Gastroenterology IRCCS “Saverio de Bellis”, Castellana Grotte, 70013 Bari, Italy; giovanni.depergola@irccsdebellis.it

**Keywords:** dietary education, mediterranean diet, dietary knowledge, type 2 diabetes mellitus, real-life study

## Abstract

**Background.** It is unclear if dietary education may increase adherence to the Mediterranean diet (MD). **Study aim.** We estimated the effect of dietary counseling on nutritional knowledge and adherence to MD in T2D adult patients. **Methods.** T2D patients who attended the Diabetology Center of the Grande Ospedale Metropolitano Niguarda were recruited (April to September 2019) and categorized into two groups: the intervention group (IG), receiving a 2.5-h education, and the control group (CG). The Moynihan questionnaire and the Mediterranean Diet Adherence Assessment Questionnaire (MDAAQ) were administered to estimate the overall knowledge and adherence to MD at baseline (T0), 1 week (T1), and 1 month (T2) later. **Results.** Seventy-two individuals (69.5 ± 8.6 years old) were included in the IG, and 52 (67.7 ± 9.2 years old) were included in the CG. All patients had sufficient dietary knowledge and intermediate adherence to MD at baseline. Those assigned to the IG showed a significant reduction in the Moynihan score from T0 (24.9 ± 2.6) to T1 (20.3 ± 1.8; *p* < 0.001) and T2 (20.4 ± 2.2; *p* < 0.001). CG had the same Moynihan score as IG individuals at T0 (24.8 ± 1.8), but their dietary knowledge was unchanged at T1 and T2 (24.9 ± 1.8). MD adherence was similar at each time in the IG, with a MDAAQ score of 4.4 ± 1.7 (T0), 5.1 ± 1.7 (T1), and 5.3 ± 1.8 (T2), and in the CG (T0: 5.1 ± 1.7; T1: 5 ± 1.5; T2: 5.1 ± 1.5). **Discussion and Conclusions.** The 2.5-h dietary counseling session improves dietary knowledge, but it is not enough to improve adherence to MD.

## 1. Introduction

About 540 million people live with diabetes worldwide, and it is expected that this number will rise to 640 and 750 million by, respectively, 2030 and 2040 [1]. T2D is the most common cause of diabetes worldwide and is responsible for more than 6 million deaths a year, also with a remarkable weight in terms of social and health costs [1]. More than 60 million individuals live with T2D in Europe [1]. The prevalence of T2D in Italy is around 6.5%, which means 3.5 to 4 million people, with relevant differences in social and economic factors and geographical distribution [2].

Other than pharmacological treatment, which plays a crucial role in T2D chronic management [3,4,5], lifestyle intervention with healthy diets and physical exercise remains the background approach to obtain and maintain adequate glucose control, prevent the onset, or reduce the risk of progression of T2D-related chronic comorbidities and complications [6,7,8]. The MD is the gold standard dietary approach in T2D because solid evidence has indicated a favorable effect on the prevention and treatment of diabetes [9], weight loss [10,11], glucose control, insulin resistance, endothelial function [12], and cardiovascular risk control [13,14,15]. Also, very low-carb diets induce rapid and effective results in terms of glucose control amelioration and weight loss in T2D and related comorbidities [16,17].

However, the effectiveness of dietary interventions depends on adequate nutritional knowledge and sufficient adherence to diet in the long term. Evidence indicates that T2D patients usually have inadequate or insufficient dietary knowledge, with negative fallouts on alimentary behaviors and adherence to dietary prescriptions [18,19,20]. Particularly, T2D patients are exposed to rich in sugar and fats ultra-processed foods and usually have a low fiber consumption, resulting in an unbalanced diet predisposing to significant deterioration of glucose control and weight gain and negative consequences on the overall T2D chronic management.

Adequate dietary education is essential to correct unhealthy nutritional behaviors in T2D. Studies demonstrated that structured education to small groups of individuals is associated with significant clinical and metabolic parameter improvements, regardless of background pharmacological treatments [18]. At the same time, patients reported relevant progress in their dietary knowledge, general health, and quality of life [21,22,23].

Apart from specific centers and clinical settings, most T2D patients do not receive specific education in a real-life setting, especially because of a lack of time, space, and professionals. Consequently, no specific attention has been concentrated on the issue, and no evidence has been provided to demonstrate the best method for administering dietary education to improve dietary knowledge and adherence to prescribed diets in a low-cost and time-saving manner in T2D individuals.

Herein, we estimated the impact of dietary counseling, administered as a single lesson, on the overall nutritional knowledge and adherence to MD up to 1 month of follow-up in T2D individuals. The strategy was thought to maximize the effect of a single and content-rich lesson and verify its efficacy with an unbiased method.

## 2. Methods

### 2.1. Study Protocol

Patients with T2D who attended the Center of Diabetology of the Grande Ospedale Metropolitano Niguarda in Milan from April to September 2019 were recruited for this retrospective study. After verifying the inclusion criteria and ruling out the exclusion criteria, the patients willing to receive dietary counseling were included. They were categorized into two groups. The intervention group (IG) included patients who received specific dietary education. The control group (CG) included patients who refused education. Most importantly, all patients received a tailored dietary (MD) plan and a comprehensive explanation of diet details from qualified dieticians after the visit.

Demographic, anthropometric, clinical, and laboratory data were collected and retrospectively analyzed from each patient. Questionnaires were administered to assess dietary knowledge and adherence to MD, and scores were collected and analyzed. Data were collected anonymously, and statistical analyses were conducted blindly due to privacy policy. Dietary knowledge and adherence to MD were retrospectively evaluated at baseline (T0), after 1 week (T1), and 1 month (T2).

### 2.2. Study Outcomes

The primary outcome was the change in the level of dietary knowledge and MD adherence after dietary counseling, compared to no education, between T0 and T1 and T0 and T2 in both groups (within-group and between-group differences).

The secondary outcomes were: (1) to assess the existence of a correlation between the level of dietary knowledge and the grade of adherence to MD at T0, T1, and T2 in both groups; (2) to analyze the effect of sex on the level of dietary knowledge and adherence to MD in both groups; (3) to analyze the effect of baseline education on the level of dietary knowledge and adherence to MD.

### 2.3. Inclusion and Exclusion Criteria

The inclusion criteria were an established diagnosis of T2D on stable background pharmacological treatment for at least 3 months, age ≥ 18 years old, and glycated hemoglobin (HbA_1c_) levels < 64 mmol/mol (<8%).

The exclusion criteria were other forms of diabetes mellitus and patients who declined dietary education.

### 2.4. Study Population

Overall, 220 patients were screened for eligibility, and 180 fulfilled the inclusion criteria. Among them, 30 patients were excluded. The leading causes of exclusion were declining to participate in the course and complete the questionnaires (n = 21) and because of other reasons (unavailability to provide written informed consent to participate, ability to accomplish the course and reply to the questions, n = 9).

All patients (n = 150) received a tailored dietary (MD) plan and a comprehensive explanation of diet details from qualified dieticians after the visit to reduce background and allocation biases. Then, patients were categorized into two groups: 80 were in the IG and 70 in the CG. A total of 124 (82.7%) patients completed the study: 72 from the IG and 52 from the CG. The patients who did not complete the follow-up were 26: 8 (5.3%) from the IG and 18 (12%) from the CG. The main withdrawal reasons were failure to accomplish the follow-up visits and inability to complete or return questionnaires after 1 week (T1) or 1 month (T2).

**Figure 1:**
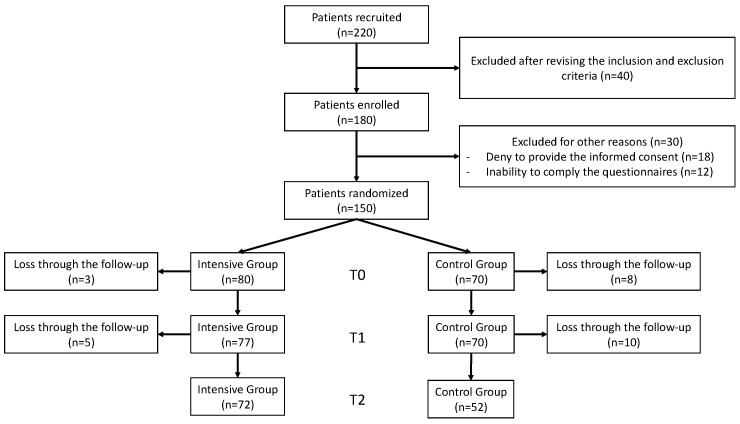
Flow diagram of included patients.

The flow chart of the study participants is depicted in Figure 1.

### 2.5. Data Collection

Demographic, clinical, anthropometric, and laboratory data were collected from the ‘Clinical Portal’ of the ASST Grande Ospedale Metropolitano Niguarda di Milano.

The leading variables were sex, age (years), duration of T2D (years), age at diagnosis of TD (years), education level, job, height (m), weight (kg), body mass index (BMI, kg/m^2^), fasting plasma glucose (FPG, mg/dL), HbA_1c_ (mmol/mol and %), serum creatinine (mg/dL), aspartate aminotransferase (AST, U/L), alanine aminotransferase (ALT, U/L), gamma-glutamyl transferase (GGT, U/L), triglycerides (mg/dL), total cholesterol (mg/dL), high-density lipoprotein (HDL) cholesterol (mg/dL), low-density lipoprotein (LDL) cholesterol (mg/dL), and non-HDL cholesterol (mg/dL).

### 2.6. Questionnaires

Questionnaires were administered to assess MD knowledge (the Moynihan questionnaire) and MD adherence (the Mediterranean Diet Adherence Assessment Questionnaire or MDAAQ). Patients received adequate instruction to fill in the questionnaires. Each questionnaire was administered at T0, T1, and T2. The questionnaires were administered individually, and the answers were collected discretely. Information was gathered, and data processing and statistical analyses were conducted anonymously.

The Moynihan questionnaire [24] represents an easy-to-use and quickly administered tool to collect information on dietary knowledge [25]. The questionnaire was administered in Italian (Supplementary Material Questionnaire S1). The Moynihan questionnaire consists of 11 questions, some of which have multiple choices, and the remaining are open-ended. Correct answers to open-ended questions are scored 1 point each, while incorrect answers score 2 points. The multiple-choice question number one has a score of 0.2 points in case of correct reply, while it is awarded 0.4 points if incorrect. For the multiple-choice questions number 4 and 6, a score of 0.1 points each is applied in case of correct answer, while 0.2 points each are awarded in case of incorrect answer. The final score, resulting from the sum of points, provides the individual’s dietary knowledge. The higher the score, the lower the level of dietary knowledge as follows: insufficient (score ≥ 26 points), sufficient (22–25 points), adequate (18–21 points), and excellent (15–17 points).

The MDAAQ represents a simple tool to assess the patient’s adherence to MD [26]. The MDAAQ comprises 15 questions that explore the frequency of food consumption characterizing the Mediterranean style. In detail, foods from questions 1 to 8 should be consumed daily, while foods from questions 9 to 15 should be consumed weekly (Supplementary Material Questionnaire S2). Adherence to MD is verified by the following leading questions, awarding one point for each condition:

- Whole-grain bread and slices ≥ 1 time/day or whole-grain pasta or rice ≥ 1–2 times/day (so either one or the other);
- Vegetables of all types (both raw and cooked) ≥ 2 times/day;
- Fruit of all types, also freshly squeezed ≥ 2 times/day;
- Olive oil for cooking and seasoning ≥ 3–4 times/day;
- Wine (white and red) for <1 time/day in women or wine (white and red) for 1–2 times/day in men;
- Red meat (beef, veal, pork), cold cuts, and sausages ≤ 1–3 times/week;
- Fish (fresh or frozen) or seafood ≥ 2–3 times/week;
- Dried fruits (walnuts, almonds, hazelnuts) ≥ 2–3 times/week;
- Legumes (chickpeas, lentils, peas, beans) ≥ 2–3 times/week.

Answers other than those herein indicated scored 0 points and are not included in the score calculation. The final score is, therefore, between 0 and 9 points, and the adherence to MD can be classified as low (0–3 points), on average (4–6 points), or high (7–9 points).

### 2.7. Dietary Education

Qualified dieticians administered dietary education to T2D patients at the Unit of Diabetology of the ASST Grande Ospedale Metropolitano Niguarda in Milan. Education consisted of a single lesson of 2.5 h during which each group of patients (3–4 individuals per group) received a detailed explanation of questionnaires, extensive theoretical education about the program’s purpose, and basic information on healthy diets and methods to implement MD adherence. In particular, the counseling focused on overall information about the relationship between energy balance and body weight with an overview of malnutrition-related diseases, the functions of macronutrients (carbohydrates, lipids, proteins) and micronutrients (vitamins and minerals), categorization of food sources of carbohydrates and identification of the most common nutrients affecting glucose levels, information about healthy distribution of nutrients in the daily diet, explanation of the importance of fibers and salt in diets, definition of MD, and specific advice on how preventing and correcting hypoglycemia for insulin-treated patients.

## 3. Statistical Analyses

Statistical analysis for clinical and anthropometric variables comparisons between the two study groups was obtained using the *t*-test for independent samples (Mann–Whitney). In contrast, the chi-square test (Fisher’s exact test) was used to analyze the nominal variables.

Differences and statistical significance of changes in mean scores of the Moynihan questionnaire and the MDAAQ were determined using the between-group and within-group for repeated measures ANOVA test.

The correlation between the mean scores of the Moynihan questionnaire and the MDAAQ was estimated using Pearson’s correlation test.

The statistical significance was set for a *p*-value < 0.05.

All statistical analyses were performed using Stata/SE 15.1 software, while graphical data processing was performed using MedCalc software 22.014.

The sample size was calculated with G Power 3.1 according to a priori analysis (two independent groups Mann–Whitney test) focused on the primary outcome. Given an expected difference between the Moynihan questionnaire scores of the IG compared to CG of at least 4 points, which was needed for IG, compared to CG, patients to pass from intermediate to good dietary knowledge due to specific education (with an assumed standard deviation of 2.5), the effect size d was 1.6. The α error was set at 0.01, and the power (1 − β) was set at 0.99. The sample size was 44 individuals (25 IG and 19 CG). Considering a drop-out rate of 20%, the sample estimation was raised to 55 individuals (31 IG and 24 CG).

## 4. Results

The two study groups’ characteristics are shown in Table 1. The IG consisted of 72 individuals (49 men and 23 women) with a mean age of 69.5 ± 8.6 years old and a duration of T2D of 13.7 ± 5.7 years old. The mean BMI was 27.9 ± 4.5 kg/m^2^, the mean FPG was 149.6 ± 29.3 mg/dL, and the mean HbA_1c_ was 57.5 ± 9.4 mmol/mol. The level of education was distributed as follows: 15 patients (20.8%) had basic education, 25 (34.7%) had intermediate education, 24 (33.3%) had high education, and eight were graduated. Furthermore, 15 individuals were found to be employed (20.8%) and 57 retired (79.2%).

The GC included 52 individuals (32 men and 20 women) with a mean age of 67.7 ± 9.2 years old and a T2D duration of 14.3 ± 7.8 years old. The mean BMI was 27.9 ± 5.2 kg/m^2^, while the mean FPG was 141.1 ± 31.2 mg/dL, and the mean HbA_1c_ was 54.2 ± 6.1 mmol/mol (7.1 ± 0.5%). Six individuals (11.5%) had basic education, 15 (28.8%) had lower-middle education, 26 (50%) had high education (50%), and 5 (9.6%) were graduates; 17 subjects were employed (32.7%) and 35 retired (67.3%).

The two study groups had similar demographic, anthropometric, clinical, and laboratory characteristics.

The mean Moynihan questionnaire scores were compared within the same group and between the two groups at T0, T1, and T2 (Table 2). Patients assigned to the IG had a baseline Moynihan questionnaire score of 24.9 ± 2.6 points, indicating sufficient dietary knowledge. After the 2.5-h dietary counseling, the overall nutritional knowledge of IG patients improved significantly after 1 week (T1), as attested by a Moynihan questionnaire score of 20.3 ± 1.8 (*p* < 0.001) and 1 month (T2, 20.4 ± 2.2 points; *p* < 0.001). Patients assigned to the CG obtained similar Moynihan questionnaire scores at T0 (24.8 ± 1.8 points) as compared to the baseline results of IG patients. The scores did not improve during the follow-up (T1 and T2, 24.9 ± 1.8), suggesting that the level of dietary knowledge was the same as compared to baseline among CG patients but lower compared to the IG group at both times (T1 and T2, *p* < 0.001).

The mean baseline MDAAQ score of IG patients was 4.4 ± 1.7, indicating an intermediate adherence to MD. Compared to T0, the scores showed a statistically significant improvement at T1 and T2, but the class of MD adherence was the same (intermediate). So, the overall MD adherence did not improve during the follow-up despite dietary counseling. Patients allocated to the CG scored the same at baseline and T1 and T2, indicating an average adherence to MD. Overall, the scores were similar between the two study groups at baseline and during the follow-up (Table 3).

**Figure 2:**
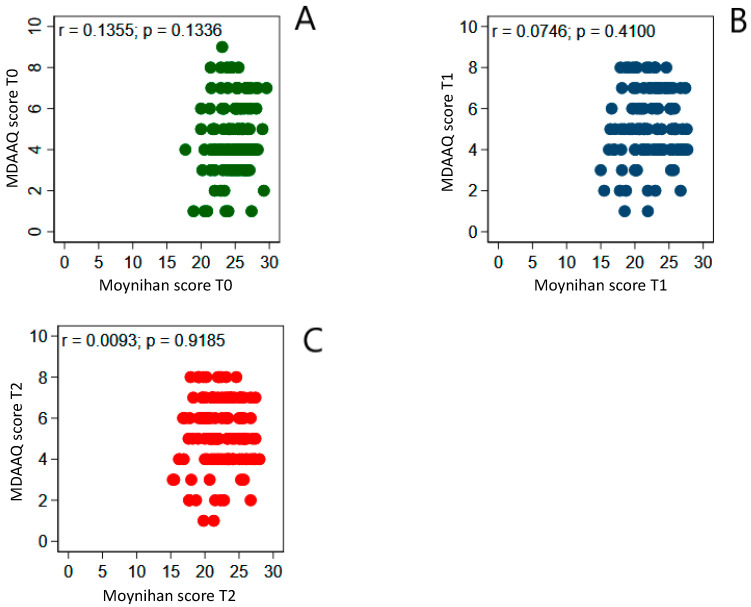
Correlation between the mean scores of the Moynihan questionnaire and the MDAAQ at T0 (**A**), T1 (**B**), and T2 (**C**) in the entire study population. Pearson’s correlation coefficient did not reach statistical significance at all times, indicating no relation between the mean level of adherence to MD and the overall dietary knowledge in the entire study population.

To better understand if the level of MD adherence was related to dietary knowledge, we correlated the mean scores obtained with the Moynihan questionnaire and the MDAAQ at baseline, T1, and T2. Pearson’s correlation coefficient did not reach statistical significance at all times (Figure 2).

In the IG, both sexes had similar levels of dietary knowledge at baseline. So, the level of dietary knowledge was sufficient (22–25 points) in men and women. All IG patients scored significantly less on the Moynihan questionnaire at T1 and T2, passing from sufficient to good dietary knowledge (18–21 points) with no sex difference (Table 4). Men and women had the same MDAAQ score at baseline, indicating sufficient adherence to MD in the IG group. At T1, the scores improved significantly in both sexes with the same magnitude and remained similar up to T2 without any statistically relevant difference (Table 4).

In the CG, both sexes had sufficient dietary knowledge at baseline and during the follow-up without any statistically relevant sex difference (Table 5).

In CG, men scored higher than women on the MDAAQ questionnaire at baseline and during the follow-up, even if the difference was not clinically and statistically significant (Table 5).

Table 6 shows a negative correlation between the education level and mean Moynihan questionnaire scores at baseline, T1, and T2. The results of this analysis suggest that the higher the level of education, the better the dietary knowledge. No correlation was observed between the level of general education and adherence to MD at baseline, T1, and T2 (Table 7).

## 5. Discussion

The pilot study presented here aimed to assess the specific effect of dietary counseling (single lesson) on the overall dietary knowledge and adherence to dietary prescription in T2D patients on stable background pharmacological treatment up to 1 month of follow-up. The education was organized following the “Group Care” model by Trento et al. [21], but it was administered once with a short follow-up to specifically reduce biases and better estimate the effect of a single bout of education. In fact, IG patients received a small-group (3–4 individuals) single bout (2.5 h) dietary counseling, while CG patients did not receive specific education or advice. Dietary counseling was administered in two sections: the first was dedicated to questionnaire delivery and explanation (the Moynihan questionnaire and MDAAQ); the second focused on dietary education. The Moynihan questionnaire and MDAAQ assessed dietary knowledge and MD adherence, respectively. Scores were calculated at baseline (T0), after 1 week (T1), and 1 month (T2) and gathered for statistical analyses. The study outcomes were to compare the levels of dietary knowledge and adherence to MD of T2D patients before and after dietary education and between patients receiving education and those not receiving education. Moreover, we tested the correlation between dietary knowledge and adherence to MD at baseline (T0), T1, and T2 and the effect of sex and general education on dietary knowledge and adherence to MD.

Our study population included patients aged 68 years old on average, with suboptimal glucose control and moderate weight excess. IG patients ameliorated dietary knowledge 1 week and 1 month after the intervention (dietary counseling) compared to baseline and compared to CG patients. Overall, these passed from sufficient to good dietary knowledge after education. The results of our study confirm existing evidence indicating that most T2D patients usually have low or sufficient dietary knowledge and require targeted nutritional education to overcome the gap [27,28]. Specific education, administered to small groups of individuals in the long term, improves dietary knowledge, as suggested by other authors [21]. Similar findings were reported by Wang et al., who demonstrated the efficacy of 6-month nutritional education on dietary knowledge and practice [29]. Our findings demonstrate that a single education bout is enough to significantly improve nutritional knowledge in T2D individuals with sufficient baseline dietary knowledge.

Nevertheless, our findings demonstrate that dietary counseling is not enough to improve adherence to MD after a single lesson on dietary education, highlighting the existence of a disagreement between the patient’s theory and practice. These findings did not align with other observations, reporting a direct association between dietary knowledge level and diet adherence [30,31,32]. Other than methodological differences between our data and other reports, a wide range of factors can explain the discrepancy, including economic, social, and familial factors, well-established and deep-seated nutritional behaviors of our participants (mostly aged), need to be guided during grocery shopping, inadequate cooking expertise, gender, and education. All these causes are difficult to be explored and could be the objective of further investigation. However, we tested some of these hypotheses. For instance, sex did not affect the level of dietary knowledge and adherence to MD before and after the observation in both groups, although some authors found that women, compared to men, were usually more interested in healthy nutrition and exhibited greater engagement in controlling their body weight with diets, better nutritional behavior, approach to nourishment, approach to the place of meal consumption, and more accurate selection of the sources of nutritional knowledge, but are also more exposed to detrimental nutritional behaviors due to psychosocial factors [33,34]. About the level of patient education, the higher the level of background instruction, the lower the Moynihan questionnaire score, and the better the dietary knowledge. Our results confirmed the findings of other groups that conducted studies among the general population and patients with T2D [35,36,37]. These data suggest that personal education and instruction positively affect dietary knowledge and potentially improve the quality of food consumption and the aptitude to follow healthy nutritional behaviors. However, the difference disappeared when comparing the levels of dietary knowledge between T1 and T0 and T2 and T0 as the result of specific dietary education. Other authors demonstrated, to support our findings, that population-based dietary education, especially in young individuals, and specific disease-related dietary education programs resulted in adequate measures to ameliorate the knowledge on diets, the quality of nutrients, and the effect of diets on general health [38,39].

As the last point, nutrition education should be associated with behavior change strategies to convert theoretical into practical results. This approach requires time and expertise. Effective behavior change strategies aim to improve dietary patterns toward healthier dietary choices and require individual intervention and social and family support. For instance, these strategies include cooking classes, meal planning workshops, and practical exercises enhancing culinary and meal preparation skills, allowing the patients (and caregivers) to be more likely to make healthier food choices. Group training also creates a sense of responsibility, and participants are prone to share their experiences, limits, and efforts, fostering motivation for dietary improvements collectively. Behavior change strategies also require dietician supervision to set specific goals, self-monitoring strategies, and manage emotional and non-emotional triggers for unhealthy eating behaviors [40,41,42].

## 6. Study Strengths and Limitations

The study’s strengths are in its methodology, including the selection of patients, sample size, standardization of procedures, and reliability of methods of outcome estimation. Moreover, the single lesson on dietary education and the short-term follow-up allowed us to precisely analyze dietary education’s effects on the primary study outcome by removing possible interfering biases.

The study’s limitations are related to the short follow-up, which was insufficient to assess the effect of specific dietary education on glucose control, body weight, and other metabolically related outcomes. Moreover, due to a lack of specific data, we did not include some variables potentially affecting the results, such as the patient’s income, employment status, and other variables that could have modulated the effectiveness of dietary education in our cohort.

## 7. Conclusions

Dietary education plays a crucial role in improving dietary knowledge and represents an essential step in the management of outpatients with T2D. A single dietary counseling, administered in 2.5 h to small groups of patients, is sufficient to achieve the goal. Therefore, our data support the effectiveness of dietary counseling in T2D patients to improve dietary knowledge, particularly in those with low background education, poor nutritional knowledge, and relevant unhealthy dietary patterns. Ideally, free of charge, online and open dietary courses would be administered by qualified centers to patients and relatives before opening them up to a broader audience.

Single dietary counseling is inadequate to improve adherence to specific diet programs (the MD in our study); hence, more investigation and specific interventions are needed to demonstrate the best cost-effective and time-saving method to improve adherence to MD. Nutritional behaviors are well-established and deep-seated, especially in old T2D patients. So, background dietary education needs to be supported along with a specific assessment of barriers complicating patients’ adherence to nutritional guidelines, including social, economic, and familial factors, and strategies to overcome the burdens to finally improve MD adherence.

## Figures and Tables

**Table 1:** Baseline characteristics of the two study groups and comparisons between the two study groups.

Baseline Characteristics		Intensive Group (n = 72)	Control Group (n = 54)	*p*-Value
Sex	Men	32 (61.5%)	49 (68.1%)	0.57
	Women	20 (38.5%)	23 (31.9%)	
Age (years)		69.5 ± 8.6	64.7 ± 9.2	0.22
Diabetes duration (years)		13.7 ± 5.7	14.3 ± 7.8	0.58
Body weight (kg)		77.6 ± 14.3	79.8 ± 16.6	0.48
BMI (kg/m^2^)		27.9 ± 4.5	27.9 ± 5.2	0.94
FPG (mg/dL)		149.6 ± 29.3	141.1 ± 31.2	0.21
HbA_1c_ (mmol/mol)		57.5 ± 9.4	54.2 ± 6.1	0.18
Creatinine (mg/dL)		0.9 ± 0.3	0.9 ± 0.3	0.55
AST (U/L)		21.8 ± 7.7	22.2 ± 5.3	0.08
ALT (U/L)		23.4 ± 9.7	23.3 ± 10.8	0.38
GGT (U/L)		26.7 ± 16.1	22.9 ± 10.1	0.36
Triglycerides (mg/dL)		139.7 ± 80.1	138.2 ± 56.4	0.44
Total cholesterol (mg/dL)		159.6 ± 30.2	169 ± 33.9	0.08
HDL Cholesterol (mg/dL)		48.7 ± 13.9	48.3 ± 13.6	0.84
LDL Cholesterol (mg/dL)		83.9 ± 25.1	93.9 ± 32.1	0.16
Non-HDL Cholesterol (mg/dL)		110.9 ± 28.2	120.6 ± 31.4	0.07
Education	Elementary	6 (11.5%)	15 (20.8%)	0.14
	Middle school	15 (28.8%)	25 (34.7%)	
	High school	26 (50%)	24 (33.3%)	
	Graduation	5 (9.6%)	8 (11.1%)	
Job-status	Work	17 (32.7%)	15 (20.8%)	0.15
	Retired	35 (67.3%)	57 (79.2%)	

Abbreviations: BMI, body mass index; FPG, fasting plasma glucose; HbA_1c_, glycated hemoglobin; AST, aspartate transaminase; ALT, alanine transaminase; GGT gamma-glutamyl transferase; HDL, high-density lipoprotein; LDL, low-density lipoprotein.

**Table 2:** Comparisons for repeated within-group and between-group measurements of the Moynihan questionnaire scores.

Moynihan Questionnaire	Intensive Group Scores Mean (±SD)	Within-Group *p*-Value	Control Group Scores Mean (±SD)	Within-Group *p*-Value	Inter-Groups *p*-Value
T0	24.9 ± 2.6	-	24.8 ± 1.8	-	ns
T1	20.3 ± 2.2	<0.001	24.9 ± 1.8	ns	<0.001
T2	20.4 ± 2.2	<0.001	24.8 ± 1.8	ns	<0.001

Moynihan questionnaire scores at baseline (T0) and after 1 week (T1) and 1 month (T2) of follow-up. Scores are reported as mean (±standard deviation) and compared through the ANOVA for repeated measurements within each group and between the two study groups. Abbreviations: SD, standard deviation.

**Table 3:** Comparisons for repeated measurements, within-group and between groups, of the MDAAQ scores.

MDAAQ Questionnaire	Intensive Group Scores Mean (±SD)	Within-Group *p*-Value	Control Group Scores Mean (±SD)	Within-Group *p*-Value	Inter-Groups *p*-Value
T0	4.4 ± 1.7	-	5.1 ± 1.7	-	ns
T1	5.1 ± 1.7	<0.001	5 ± 1.5	ns	ns
T2	5.3 ± 1.8	<0.001	5.1 ± 1.5	ns	ns

MDAAQ questionnaire scores at baseline (T0) and after 1 week (T1) and 1 month (T2) of follow-up. Scores are reported as mean (±standard deviation) and compared through the ANOVA for repeated measurements within each group and between the two study groups. Abbreviations: SD, standard deviation.

**Table 4:** Comparison between the Moynihan questionnaire and MDAAQ scores of IG patients at T0, T1, and T2.

**Moynihan** **Questionnaire**	**Men Scores** **Mean (±SD)**	**Within-Group** ***p*-Value**	**Women Scores Mean (±SD)**	**Within-Group** ***p*-Value**	**Inter-Groups** ***p*-Value**
T0	24.9 ± 2.2	-	25.1 ± 2.4	-	ns
T1	20.5 ± 2.1	<0.001	20.1 ± 2.1	<0.001	ns
T2	20.8 ± 2.3	<0.001	19.9 ± 1.9	<0.001	ns
**MDAAQ** **Questionnaire**	**Men Scores** **Mean (±SD)**	**Within-Group** ***p*-Value**	**Women Scores** **Scores Mean (±SD)**	**Within-Group** ***p*-Value**	**Inter-Groups** ***p*-Value**
T0	4.3 ± 1.8	-	4.7 ± 1.6	-	<0.001
T1	4.5 ± 1.7	ns	5.4 ± 1.9	ns	<0.001
T2	4.7 ± 1.8	ns	5.5 ± 1.7	ns	<0.001

Mean scores of the Moynihan questionnaire of IG patients were similar in both sexes at baseline and lessened at T1 and T2, indicating an overall improvement of dietary knowledge in both sexes. Mean MDAAQ scores were similar in both sexes at each time point, indicating that the patient’s adherence to MD did not improve after the dietary education. However, women compared to men scored higher at every time (*p* < 0.001) even if the difference was not clinically relevant since the level of adherence to MD was the same (sufficient adherence) as baseline.

**Table 5:** Comparison between the Moynihan questionnaire and MDAAQ scores of GG patients at T0, T1, and T2.

**Moynihan** **Questionnaire**	**Men Scores** **Mean (±SD)**	**Within-Group** ***p*-Value**	**Women Scores Mean (±SD)**	**Within-Group** ***p*-Value**	**Inter-Groups** ***p*-Value**
T0	25.1 ± 1.9	-	24.3 ± 2.4	-	ns
T1	25.1 ± 2	ns	24.4 ± 2.3	ns	ns
T2	25.1 ± 2	ns	24.3 ± 2.3	ns	ns
**MDAAQ** **Questionnaire**	**Men Scores** **Mean (±SD)**	**Within-Group** ***p*-Value**	**Women Scores** **Scores Mean (±SD)**	**Within-Group** ***p*-Value**	**Inter-Groups** ***p*-Value**
T0	5.3 ± 1.6	-	4.6 ± 2.1	-	ns
T1	5.2 ± 1.3	ns	4.6 ± 1.9	ns	ns
T2	5.3 ± 1.3	ns	4.6 ± 1.8	ns	ns

Mean scores of the Moynihan questionnaire of CG patients were similar in both sexes at baseline and over the follow-up (T1 and T2 compared to T0, *p* = 0.157), indicating that dietary knowledge was steady without specific education. Mean scores of the MDAAQ of CG patients were similar in both sexes at baseline and T1 and T2 (*p* = 0.192), indicating that patient adherence to MD was the same during the entire study period. Although men, compared to women, scored higher every time, the result was not statistically and clinically relevant (*p* = 0.626).

**Table 6:** Correlation between the level of education and dietary knowledge.

Time	Independent Variable	Dependent Variable	Sample Size	Correlation Coefficient	*p*-Value
T0	Education level	Moynihan questionnaire score	124	r = −0.58	<0.001
T1	Education level	Moynihan questionnaire score	124	r = −0.26	0.003
T2	Education level	Moynihan questionnaire score	124	r = −0.28	0.002

Pearson’s correlation test was used to analyze the correlation between the level of education and dietary knowledge, which was estimated using the Moynihan questionnaire. An inverse correlation was found each time, suggesting that higher education was associated with better dietary knowledge.

**Table 7:** Correlation between the level of education and adherence to the Mediterranean diet.

Time	Independent Variable	Dependent Variable	Sample Size	Correlation Coefficient	*p*-Value
T0	Education level	MDAAQ score	124	r = 0.16	0.86
T1	Education level	MDAAQ score	124	r = −0.11	0.23
T2	Education level	MDAAQ score	124	r = −0.10	0.26

Pearson’s correlation test to analyze the correlation between the level of education and adherence to the Mediterranean diet estimated with the MDAAQ. No correlation was found at each time, suggesting that the level of education does not affect the adherence to the Mediterranean diet. Abbreviation: MDAAQ, Mediterranean Diet Adherence Assessment Questionnaire.

## Data Availability

The data presented in this study are available on reasonable request from the first and corresponding authors.

## Supplementary Materials

The following supporting information can be downloaded at: https://www.mdpi.com/article/10.3390/nu17071139/s1, Questionnaire S1: Moynihan questionnaire. Questionnaire S2: MDAAQ.

## List of Abbreviations

ASpartate aminotransferase	AST
ALanine aminotransferase	ALT
Body Mass Index	BMI
Control Group	CG
Fasting Plasma Glucose	FPG
Gamma-Glutamyl Transferase	GGT
Glycated hemoglobin	HbA_1c_
High-Density Lipoprotein	HDL
Intervention Group	IG
Low-Density lipoprotein	LDL
Mediterranean Diet	MD
Mediterranean Diet Adherence Assessment Questionnaire	MDAAQ
Type 2 Diabetes	T2D
