# Supplementary material for: A Single Lesson on Dietary Education Improves Dietary Knowledge in Adults with Type 2 Diabetes: A Real-Life Monocentric Italian Study
Source: Nutrients. 2025 Mar 26;17(7):1139. doi: 10.3390/nu17071139 (PMC11990264; doi:10.3390/nu17071139)
Supplement: Supplementary file 1 [file nutrients-17-01139-s001.zip › Supplementary material Questionnaire S1.pdf]

APPENDICE :
versione italiana del questionario sulle conoscenze alimentari

Alle risposte esatte verrà assegnato un punteggio di 1, a quelle errate di 2. Per la domanda a risposta multipla numero uno il punteggio sarà : di 0,2 per ogni risposta esatta e 0,4 per ogni risposta errata. Per le domande a risposta multipla numero 4 e 6 il punteggio sarà: di 0,1 per ogni risposta esatta e 0,2 per ogni risposta errata.

1) Il sottostante grafico rappresenta le proporzioni di cibo raccomandato che dovremmo introdurre nella nostra dieta per raggiungere un equilibrio ideale tra i diversi cibi.

Le sezioni del grafico rappresentano ognuna uno dei 5 gruppi di alimenti seguenti.

1 - frutta e verdura

2 - pane, altri cereali e patate

3 - latte, latticini e formaggi

4 - grassi e alimenti contenenti zuccheri in grande quantità

5 - carne, pesce, uova e legumi

Quale sezione ritieni che rappresenti ciascuno di questi gruppi di alimenti? Scrivi il numero del gruppo degli alimenti nella sezione che tu ritieni rappresenti quel gruppo.

Per esempio se pensi che la sezione A è una buona rappresentazione di quanto latte e latticini dovremmo mangiare, metti 3 dentro la sezione A.

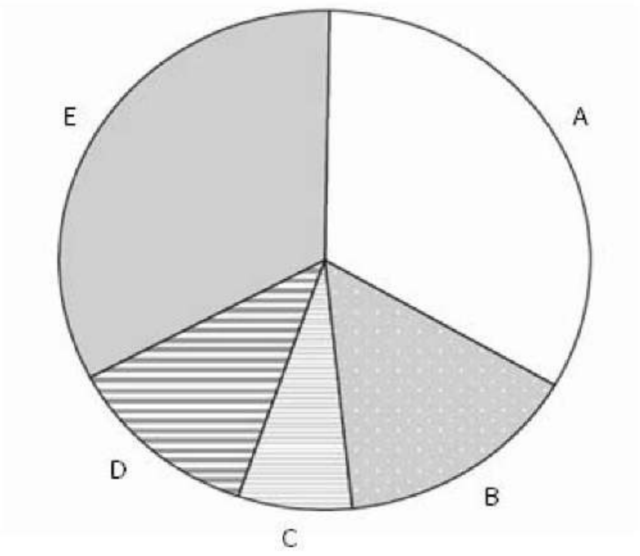

GICR-IACPR
Italian Association for Cardiovascular Prevention, Rehabilitation and Epidemiology

ANDID
associazione nazionale dietisti

2) Quante porzioni di frutta e verdura pensi che gli esperti consiglino di consumare ogni giorno? (per esempio una porzione potrebbe essere una mela o un piattino di carote grattate)

☐ 0-1 ☐ 2-3 ☐ 4-5 ☐ 5 o più ☐ non lo so

3) Quali grassi gli esperti dicono sia più importante diminuire nell'alimentazione?

☐ grassi monoinsaturi

☐ grassi polinsaturi

☐ grassi saturi

☐ tutti i grassi

☐ non lo so

4) Quali cibi pensi che gli esperti della nutrizione raccomandano di consumare in minore o maggiore quantità? (segna ✓ in solo una casella per ciascun cibo)

	Molto	Abbastanza	Poco	Non lo so
verdura				
cibi zuccherati (bibite, dolci)				
formaggi				
carne rossa				
carboidrati (pasta, pane, patate)				
cibi grassi (fritture, patatine, maionese)				
cibi ricchi di fibra				
frutta				
pesci grassi				
cibi salati				

GICR-IACPR
Italian Association for Cardiovascular Prevention, Rehabilitation and Epidemiology

ANDID
associazione nazionale dietisti

5) Quali sono le principale fonti di grassi omega 3 ?(puoi segnarne più di uno)

- ☐ merluzzo
- ☐ aringa
- ☐ una porzione di bastoncini di pesce surgelati
- ☐ salmone
- ☐ granchio
- ☐ sgombro
- ☐ non lo so

6) Quali tra i seguenti cibi pensi che contenga un alta o una bassa quantità di fibra (segna ✓ solo in una casella per ciascun alimento)

	Rilevante	Non Rilevante	Non lo so
uova			
carne rossa			
noci			
pesce			
funghi			
pollo			
fagioli			
pane integrale			
frutta			
verdura			

GICR-IACPR
Italian Association for Cardiovascular Prevention, Rehabilitation and Epidemiology

ANDID
associazione nazionale dietisti

7) Quale panino pensi sia più salutare?

☐ due sottili fette di pane e una spessa fetta di formaggio

☐ due spesse fette di pane e una sottile fetta di formaggio

☐ uguali

8) Quale è la scelta migliore per un pranzo leggero, povero di grassi e ricco di fibre?

☐ pollo arrosto

☐ pane integrale con formaggio

☐ pasta e fagioli

☐ torta salata al formaggio

☐ non lo so

9) Che cosa pensi sia più salutare?

☐ una porzione abbondante di pasta con poco sugo di carne

☐ una piccola porzione di pasta con abbondante sugo di carne

☐ non lo so

10) Se una persona vuole ridurre i grassi della dieta, quale dovrebbe essere la scelta migliore tra i seguenti alimenti?

☐ bistecca alla griglia

☐ salsicce alla griglia

☐ fesa di tacchino alla griglia

☐ braciola di maiale alla griglia

☐ non lo so

VALIDAZIONE DELLA VERSIONE ITALIANA DEL QUESTIONARIO DI MOYNIHAN SULLE CONOSCENZE ALIMENTARI. Pagina 5

GICR-IACPR
Italian Association for Cardiovascular Prevention, Rehabilitation and Epidemiology

ANDID
associazione nazionale dietisti

11) Sei consapevole di tutti i problemi e le malattie legate a un basso consumo di fibra alimentare?

☐ sì ☐ no ☐ non lo so

Se sì , quali problemi di salute o malattie pensi siano dovute a un basso consumo di fibra alimentare?

---

11 a) Sei consapevole dei vantaggi per la salute dell'uso di frutta e verdura ?

☐ sì ☐ no ☐ non lo so

Se sì, quali problemi di salute sono correlati a un basso consumo di frutta e verdura ?

---

11 b) Sei consapevole dei problemi di salute correlati alla quantità di grassi consumati?

☐ sì ☐ no ☐ non lo so

Se sì, quali problemi di salute sono correlati ai grassi?

---

11 c) Sei consapevole dei problemi di salute correlati alla quantità di zuccheri consumati?

☐ sì ☐ no ☐ non lo so

Se sì, quali problemi di salute sono correlati al consumo di zuccheri?

---

11 d) Sei consapevole dei problemi di salute correlati al quantitativo di sale consumato ?

☐ sì ☐ no ☐ non lo so

Se sì, quali problemi di salute sono correlati al consumo del sale?
