# Supplementary material for: A Single Lesson on Dietary Education Improves Dietary Knowledge in Adults with Type 2 Diabetes: A Real-Life Monocentric Italian Study
Source: Nutrients. 2025 Mar 26;17(7):1139. doi: 10.3390/nu17071139 (PMC11990264; doi:10.3390/nu17071139)
Supplement: Supplementary file 1 [file nutrients-17-01139-s001.zip › Supplementary material Questionnaire S2.pdf]

NOME E COGNOME ____________________

ID______________ **Data di nascita** ____/____/_____ **Data di compilazione**_____/_____/______

Peso __________ **kg** - **Altezza** __________ **cm**

Buongiorno, Vorremmo farle alcune domande sulla sua dieta abituale. Indichi, con una crocetta "X", il numero di porzioni normalmente consumate per i 15 alimenti o gruppi di alimenti elencati nella tabella sottostante.

Si aiuti con le porzioni di riferimento per identificare la sua frequenza di consumo giornaliera, per gli alimenti elencati dalla domanda n. 1 alla n. 8 e la sua frequenza di consumo settimanale per gli alimenti dalla n. 9 alla n. 15.

Se abitualmente consuma una porzione molto piccola o molto grande (rispetto alla porzione di riferimento) dimezzi o raddoppi la frequenza di consumo. Per esempio se normalmente beve mezzo litro di vino al giorno (corrispondenti a circa 4 bicchieri), la frequenza da segnare in tabella sarà "3-4" porzioni al giorno.

E' molto importante che risponda a tutte le domande. Nel caso non consumasse qualche alimento, ricordi di fare una crocetta su "mai o raramente".

Con quale frequenza consuma normalmente una porzione dei seguenti alimenti?

**ALIMENTI**	**PORZIONE**	**FREQUENZA DI CONSUMO AL GIORNO**				
		**Mai o raramente**	**Meno di 1 volta /giorno**	**1 volta /giorno**	**2 volte /giorno**	**≥ 3 volte /giorno**
1. Pasta o riso di tipo integrale	**80 gr**	☐	☐	☐	☐	☐
2. Verdura tutti i tipi (sia cruda che cotta)	**200 gr (80 gr insalata)**	☐	☐	☐	☐	☐
3. Frutta tutti i tipi, anche la spremuta fresca	**150 gr**	☐	☐	☐	☐	☐
4. Latte e yogurt	**1 bicchiere/ vasetto (125 gr)**	☐	☐	☐	☐	☐
		**Mai o raramente**	**Meno di 1 volta /giorno**	**1-2 volte /giorno**	**3-4 volte /giorno**	**≥ 5 volte /giorno**
5. Pane e fette di tipo integrale	**1-2 fette (50 gr)**	☐	☐	☐	☐	☐
6. Olio di oliva per cucinare e condire	**1 cucchiaio (10 ml)**	☐	☐	☐	☐	☐
7. Burro, margarina o panna da cucina per cucinare	**1 noce (10 gr)**	☐	☐	☐	☐	☐
8. Vino (bianco e rosso)	**1 bicchiere (125 ml)**	☐	☐	☐	☐	☐
**ALIMENTI**	**PORZIONE**	**FREQUENZA DI CONSUMO ALLA SETTIMANA**				
		**Mai o raramente**	**Meno di 1 volta /sett**	**1-3 volte /sett**	**4-6 volte /sett**	**≥ 7 volte /sett**
9. Carne rossa (bovino, vitello, maiale), affettati e salumi	**100 gr (carne) 50 gr (salumi)**	☐	☐	☐	☐	☐
10. Carne bianca (pollo, tacchino, coniglio)	**100 gr**	☐	☐	☐	☐	☐
11. Bevande dolci o gassate (tipo coca-cola, aranciata, gassosa, ecc)	**1 bicchiere (200 ml)**	☐	☐	☐	☐	☐
12. Dolci o pasticcini (non fatti in casa), come torte, biscotti, creme o dolci al cucchiaio	**100 gr**	☐	☐	☐	☐	☐
		**Mai o raramente**	**Meno di 1 volta /sett**	**1 volta /sett**	**2-3 volte /sett**	**≥ 4 volte /sett**
13. Pesce (fresco o surgelato) o frutti di mare	**150 gr (pesce) 50 gr (frutti di mare)**	☐	☐	☐	☐	☐
14. Frutta secca (noci, mandorle, nocciole)	**1 pugno (30 gr)**	☐	☐	☐	☐	☐
15. Legumi (ceci, lenticchie, piselli, fagioli)	**50 gr (secchi) 150 gr (scatola / freschi)**	☐	☐	☐	☐	☐
